# Reply to Zadegan et al.: Spatial dependence is real, but not every correction travels well

**DOI:** 10.1073/pnas.2613518123

**Published:** 2026-05-18

**Authors:** Jonas R. Kunst, Tomasz Besta, Milan Obaidi

**Affiliations:** ^a^ Department of Communication and Culture, BI Norwegian Business School, Oslo 0484, Norway; ^b^Institute of Psychology, University of Gdańsk, Gdańsk 80-309, Poland; ^c^ Department of Psychology, University of Copenhagen, 1353 Copenhagen, Denmark

We welcome Zadegan et al.’s commentary (1) concerning our recent paper (2). We agree that spatial dependence is a legitimate methodological consideration in cross-national research (3).

The commentary replicates most of our bivariate country-level findings. The Democracy Index, Human Development Index, and GDP per capita all retain statistical significance in the Spatial Durbin Model (table 1 in ref. 1). This convergence strengthens our conviction that these factors covary with reduced offensive extremism.

However, several reanalysis features undermine the commentary’s evidentiary value. First, the bivariate Global Terrorism Index model drops to nonsignificance under spatial adjustment. Yet, terrorism is inherently regional and geographically clustered. Partialing out the terrorism’s spatial lag likely absorbs the very substantive variance of interest. Geographic clustering of conflict is not merely a statistical nuisance but a core feature of the ecology of violence that calibrates offensive violent extremist intentions.

Second, the authors claim cross-border spillovers in which neighboring-country wealth and inequality predict lower offensive intentions. These spatial lag effects are possible but may be artifactual. Because wealth and inequality are heavily regionalized globally, the spatial lag may act as a regional proxy. The theoretical mechanism by which a neighboring country’s internal inequality would influence an individual’s psychological intention to commit violence is tenuous.

Third, the spatial weights are imperfectly suited to our sample. While the commentary pairs Queen contiguity for spatial lags with inverse distance weights for error correlation, the novel spillover inferences (W × X terms) hinge on Queen contiguity. Queen contiguity assigns zero neighbors to island nations, which comprise at least seven of 58 countries, making the correction partial. The commentary states that “results hold under alternative specifications,” but neither the specifications nor coefficients are reported.

Fourth, the commentary’s spatial Durbin model is fit on 58 country-level point estimates treated as equally precise, even though these means are computed from sample sizes ranging from 72 (Nepal) to 1,097 (Italy) participants. Our multilevel framework avoids this aggregation step: It fits directly on all individual-level observations, with country-level variation modeled as a random effect that weights countries by their precision. The commentators’ approach may therefore be a loss of rigor, not a gain.

Finally, the commentary’s Model 6, which enters all six predictors simultaneously, is the source of most apparent fragility in our findings, yet it is analytically indefensible. As explicitly noted, our paper tested macrolevel predictors separately due to high intercorrelations (up to *r* = 0.79) and partly overlapping data sources. Entering these jointly into a spatial model with only 56 countries, while estimating spatial lag terms for each predictor, violates the assumption of independence of observations and introduces severe multicollinearity, yielding effectively uninterpretable estimates. The negative GTI effect on defensive VEI in Model 6 is (as the commentators acknowledge) the signature of a suppressor artifact rather than a substantive spatial finding. The proposed interpretation, that terrorism exposure may shift populations toward “protective rather than offensive orientations,” is inconsistent with our bivariate results, with the exploratory GPI subindicator analyses (*SI Appendix*, Table S13 in ref. 2), and with the broader literature linking exposure to collective violence with elevated threat-reactive responses (4).

## Data Availability

All study data are included in the article.
